# Cancer Stem Cells in Breast Cancer

**DOI:** 10.3390/cancers3011311

**Published:** 2011-03-15

**Authors:** Ryou-u Takahashi, Fumitaka Takeshita, Tomohiro Fujiwara, Makiko Ono, Takahiro Ochiya

**Affiliations:** 1 Division of Molecular and Cellular Medicine, National Cancer Center Research Institute, 1-1, Tsukiji 5-chome, Chuo-ku, Tokyo 104-0045, Japan; E-Mails: rytakaha@ncc.go.jp (R.T.); futakesh@ncc.go.jp (F.T.); tofujiwa@ncc.go.jp (T.F.); makono@ncc.go.jp (M.O.); 2 Department of Orthopedic Surgery, Okayama University Graduate School of Medicine, Dentistry, and Pharmaceutical Sciences, Okayama, Japan, 2-5-1 Shikata-cho, Okayama City, Okayama 700-8558, Japan

**Keywords:** cancer stem cells, breast cancer, EMT, TGF-β, microRNAs

## Abstract

The cancer stem cell (CSC) theory is generally acknowledged as an important field of cancer research, not only as an academic matter but also as a crucial aspect of clinical practice. CSCs share a variety of biological properties with normal somatic stem cells in self-renewal, the propagation of differentiated progeny, the expression of specific cell markers and stem cell genes, and the utilization of common signaling pathways and the stem cell niche. However, CSCs differ from normal stem cells in their chemoresistance and their tumorigenic and metastatic activities. In this review, we focus on recent reports regarding the identification of CSC markers and the molecular mechanism of CSC phenotypes to understand the basic properties and molecular target of CSCs. In addition, we especially focus on the CSCs of breast cancer since the use of neoadjuvant chemotherapy can lead to the enrichment of CSCs in patients with that disease. The identification of CSC markers and an improved understanding of the molecular mechanism of CSC phenotypes should lead to progress in cancer therapy and improved prognoses for patients with cancer.

## 1. Introduction

To overcome problems related to conventional cancer therapy, many cancer researchers focus on tumor-initiating cells (TICs) or cancer stem cells (CSCs). The term CSC is frequently used to mean TIC. Although the CSC theory is well established and widely accepted [1-4], some controversies remain. CSCs, like normal stem cells, show asymmetric cell division [5,6], chemoresistance [7], and tumorigenicity [5]. CSCs have the capacity to form secondary/tertiary tumors upon serial xenotransplantation into immunodeficient rodents and show the same features as the original tumors. The demonstration of these three capacities, the gold standard for the evaluation of CSC phenotypes, reflects the malignancy of tumors and is critical in cancer therapy. Recent studies have demonstrated that CSCs show metastatic ability. In this article, we discuss the general features of CSCs and focus on their characteristics in breast cancers, in which the identification and analysis of CSC markers and CSC phenotypes are well refined.

## 2. Origin of Cancer Stem Cells

The development of fluorescent antibodies, flow cytometry, and associated cell sorting has facilitated the identification of the cell population that initiates tumors [5,6]. Furthermore, the development of mouse strains with profound immunodeficiencies has allowed the evaluation of tumor formation ability. With these developing tools, Dick and colleagues demonstrated that, in human acute myeloid leukemia (AML), a rare malignant cell showed the ability to repopulate the entire original disease over several transplantations and that such a cell population existed within the immature CD34^+^CD38^−^, and not the CD34^+^CD38^+^, sub-population [6]. Because of the similarity between CD34^+^CD38^−^ cells and normal hematopoietic stem cells (HSCs), the origin of the cell population in which the disease arises was thought to be this normal population. Subsequent work with colony-formation assay and lentivirus vector tracking identified the cell population that was able to repopulate the disease not only over several transplantations but also over a single transplantation as quiescent stem cells [8]. This hierarchy, which closely mimics the normal process of hematopoietic precursor development, may explain the CSC biology of childhood AML [8].

## 3. Solid Tumor CSCs

The first solid CSCs were identified in breast tumors in 2003 [5]; subsequently, CSCs have been identified in brain [9,10], colon [11,12], melanoma [13,14], pancreatic [15,16], prostate [17], ovarian [18], hepatic [19], lung [20], and gastric cancers [21]. In breast cancer, CD44^+^ CD24^−/low^ ESA^+^ (epithelial surface antigen, also known as EpCAM) cells were identified as CSCs in a number of solid malignancies [5,22]. These cells are thought to share stem cell-like properties because they are capable of reconstituting the heterogeneity of the original primary tumors. The same approach was used for brain tumors exhibiting a subpopulation of CD133^+^ cells with CSC phenotypes [9,10]. CD133^+^ CSCs were subsequently isolated from colon and pancreatic cancers [23,24]. Recently, in hepatocellular carcinoma, EpCAM was identified as a CSC marker and found to be expressed in normal epithelial progenitor cells [25]. Furthermore, aldehyde dehydrogenase (ALDH) is a CSC marker in breast cancers [26]. The representative cell surface markers for human hematologic and solid cancers reported to date are listed in Table 1.

## 4. CSC Markers

### 4.1. Side Population

Following the identification of the Hoechst 33342 dye-efflux side population (SP) in bone marrow cells in mice as hematopoietic stem cells [30,31], SP cells with stem cell-like properties have been identified in a variety of human hematologic cells and solid malignant cells. These cells show CSC phenotypes characterized by asymmetric cell division, drug resistance, and tumorigenicity [16,32-34]. Thus, SP cells can be assumed to play important roles in CSC fractions. Zhou et al. demonstrated that the expression of the *ABCG2* gene, a member of the ATP binding cassette (ABC) transporter superfamily, is an important determinant of the SP phenotype [35].

A recent study pointed out the following problems in using SP cells as a CSC fraction [36]. First, cells resistant to Hoechst 33342 dye do not necessarily show tumorigenicity and metastatic ability as CSCs. For example, ABCG2-positive MCF-7 cells showed no more tumorigenic potential than did ABCG2-negative cells [37]. Second, the staining condition, staining time, and cellular concentration of Hoechst dye affect the viability of the SP fraction [36]. Third, cytometry gating strategies used to isolate SP cells lack the consistency of gating strategies used in marker staining [36]. These problems latently lead to cross-contamination of the SP and the non-SP fractions, resulting in controversial data.

### 4.2. CD34

CD34 is a monomeric cell-surface antigen with a molecular mass of approximately 110 kD [6]. In common acute lymphoblastic leukemia (CALL), the expression of CD34 is positively correlated with CD10, known as common acute lymphocytic leukemia antigen (CALLA) [38]. CD34-positive leukemic cells are thought to be a less-differentiated phenotype than CD34-negative cells [6]. CD10 is a key element in the niche that maintains the progenitor and stem cell pools in the mammary lineage [39].

### 4.3. CD44

CD44 is a useful marker for collecting CSCs not only in breast tumors but also in a variety of other tumor models [5,40,41]. CD44 may also be important in metastasis. Through the implantation of patient tumors or breast CSCs into mouse mammary fat pads and the use of noninvasive imaging strategies, it was demonstrated that CD44^+^ cells from both primary tumors and lung metastases showed high tumorigenicity [42]. In addition, CD44 variant isoforms are differentially expressed during pregnancy and involution, indicating a role in normal breast epithelial homeostasis [43]. p53 inhibits the expression of the CD44 cell-surface molecule via binding to a noncanonical p53-binding sequence in the CD44 promoter [44]. This interaction enables an untransformed cell to respond to stress-induced, p53-dependent cytostatic and apoptotic signals that would otherwise be blocked by the actions of CD44 [44].

### 4.4. CD133

As shown in Table 1, recent studies have demonstrated that CD133 (prominin-1) is a specific marker of CSCs in a wide spectrum of malignant tumors [10,12,24]. CD133 was the first identified member of the prominin family of the 5-transmembrane glycoprotein [45]. In 1997, Yin et al. produced a novel monoclonal antibody (MAb) that recognized the AC133 antigen [46], a glycosylation-dependent epitope of CD133, and the expression of AC133 restricted in CD34^+^ progenitor cells from adult blood [47]. CD133 cDNA encodes a 5-transmembrane domain molecule with an extracellular N-terminus, a cyotoplasmic C-terminus, and two large extracellular loops with eight consensus sites for *N*-linked glycosylation [48]. The characteristic feature of CD133 is its rapid downregulation during cell differentiation [49]. This feature makes CD133 a unique cell surface marker for the identification and isolation of stem cells and progenitor cells in several tissues [45]. According to the CSC theory, CSCs express some stem cell markers as normal stem cells [1,2]. Therefore, tumor cells expressing CD133 independently or in combination with other stem or progenitor cell markers are thought to represent CSCs.

### 4.5. Aldehyde Dehydrogenase (ALDH)

ALDH1 is a detoxifying enzyme responsible for the oxidation of intracellular aldehydes [50]. ALDH has been reported to have a role in the early differentiation of stem cells in the oxidization of retinol to retinoic acid [51,52]. Furthermore, high ALDH activity has been observed in murine and human hematopoietic and neural stem and progenitor cells [53,54]. An increase in ALDH activity has also been found in stem cell populations in multiple myeloma and AML [55]. Therefore, ALDH activity may provide a common marker for both normal and malignant stem and progenitor cells. In several tumors, the measurement of ALDH activity is a useful approach in the identification and isolation of CSCs [56-59]. However, the ALDEFLUOR assay does have some limitations in the isolation of the most tumorigenic population, notably in tumors of different origins. For example, both ALDEFLUOR (bright) and ALDEFLUOR (low) from the melanoma cell line were able to initiate tumors after inoculation into NOD/SCID mice [60]. Moreover, tumors generated from ALDEFLUOR (low) cells grew faster and bigger than tumors from ALDEFLUOR (bright), even among passages. These results suggest that the ALDEFLUOR-positive population in melanoma is not stem cell-enriched, unlike the ALDEFLUOR-negative population [60]. Furthermore, the stem cell population identified using the ALDEFLUOR assay is probably heterogeneous and needs to be dissected using additional markers.

### 4.6. microRNA

Despite progress in the identification of CSC markers, the CSC theory has been complicated by a lack of clearly defined developmental surface markers specific for individual tumor types, making the molecular mechanism of common phenotypes in CSC fractions the object of much ongoing research. In this regard, microRNAs (miRNAs), as a part of non-coding RNA, are considered to be novel markers for CSCs, as shown in Table 2.

## 5. Breast Cancer Stem Cells (BCSCs)

### 5.1. Identification of BCSCs

The pioneering study by Clarke and colleagues used breast cancer xenografts to isolate a population of cells able to initiate tumors in NOD/SCID mice [5]. This population was defined by the combined expression of the cell surface marker CD44^+^/CD24^−/low^/lin^−^. As few as 200 of these cells generated tumors in NOD/SCID mice, whereas 20,000 cells that did not display this phenotype failed to do so. The NOD/SCID tumors recapitulated the entire heterogeneity of the initial tumor. Furthermore, the CD44^+^/CD24^−/low^/lin^−^ cell population was able to reinitiate tumors in NOD/SCID mice and retained this ability after serial passages. Thus, these cells, which had the ability to self-renew and to differentiate and which displayed tumorigenic capacity, had CSC features.

Through the use of the CD44^+^/CD24^−/low^/lin^−^ phenotype and another marker, the ALDEFLUOR assay, it has been shown that cells able to initiate tumors in mice are among the ALDEFLUOR-positive cells, the cells displaying both phenotypes being the most tumorigenic, and that none of the CD44^+^/CD24^−/low^/lin^−^ cells without ALDEFLUOR activity can grow in mice [26]. These results indicate that the CD44^+^/CD24^−/low^/lin^−^ population contains some but not all of the CSCs in breast tumors. Moreover, while CD44 appears to be a common stem cell marker [73-75], as well as a promising therapeutic target [76,77], the CD44^+^/CD24^−/low^/lin^−^ phenotype is probably tissue restricted. For example, pancreatic cancer cells with stem cell properties of self-renewal, the ability to produce differentiated progeny, and increased expression of the developmental signaling molecule sonic hedgehog display a CD44^+^/CD24^+^/ESA^+^ phenotype [16,78].

### 5.2. Epithelial-Mesenchymal Transition (EMT)

Pathophysiological conditions such as tissue injury or tumorigenesis can trigger differentiated cells to acquire a multipotent stem cell-like phenotype through EMT induction [32,79]. This acquisition may mirror developmentally regulated EMT signaling pathways, such as Wnt, Notch, and Hedgehog, which drive both normal and CSC renewal and maintenance [32,80,81]. Metastatic cancer cells, which have presumably undergone EMT, may exhibit CSC phenotypes. For instance, in murine and human melanoma, cancer metastasis is accelerated through immunosuppression during Snail-induced EMT [82]. A recent study revealed a subpopulation of CD26^+^ cancer stem cells showing not only chemoresistance but also metastatic potential in human colorectal cancer [27]. In breast tumors, CD44^+^ cells from both primary tumors and lung metastases are highly enriched for tumor-initiating cells [42].

Empirical evidence connecting EMT to the acquisition of stem cell phenotypes has recently been reported by Weinberg et al. [83]. The differentiated mammary epithelial cells that have undergone EMT either on transforming growth factor beta (TGF-β) treatment or by the expression of E-cadherin transcriptional repressors, such as Snail or Twist, generate CD44^+^/CD24^−^ cells as BCSCs [83]. The same researchers also demonstrated that E-cadherin knockdown generated CD44^+^/CD24^−^ cells showing BCSC properties [7]. In addition, stem cells isolated from normal breast tissue or breast cancers express a number of canonical EMT markers [83]. Of clinical significance, an immune response that induces the EMT-associated emergence of CSCs as CD8^+^T-cells can induce the dedifferentiation of breast cancer cells, resulting in the generation of CD44^+^/CD24^−^ stem cell-like cells [84]. In summary, accumulating evidence links EMT to the generation of CSC-like phenotypes, which may be prerequisites for cancer cell metastasis.

### 5.3. TGF-β

TGF-β family cytokines are mediators of embryonic development and tissue homeostasis in adults [85]. TGF-β signaling controls many types of physiological and pathophysiological EMT [79,86]. Type I and type II TGF- β receptors (TβRI and RII, respectively) are dual specificity kinases that possess both serine/threonine and tyrosine kinase activities [87]. TβRI activates effector Smads 2 and 3, which subsequently bind to a co-smad, Smad4. The Smad2, Smad3, and Smad4 complexes can associate with accessory transcription factors to activate the expression of target genes that cause changes in cellular differentiation. Notably, Smads associate with zinc finger E-box binding (ZEB) proteins to repress the expression of E-cadherin during the initiation of EMT [88-91]. TGF-β suppresses tumor growth strongly and induces apoptotic effects during the early stages of tumor progression. However, tumors show that EMT becomes insensitive to TGF-β-mediated growth inhibition while showing increased tumor invasion and metastasis. Significant progress has been made in the elucidation of the cellular and molecular events that regulate tumor-promoting effects on microenvironments and tumor cells. Indeed, in breast cancer, mesenchymal stem cell (MSC)-derived TGF-β1 increases the frequency of regulatory T cells, resulting in the support of breast cancer growth [92]. TGF-β signaling is also reported to regulate the expression and activity of matrix metalloproteases (MMP-2 and MMP-9) and downregulate the expression of the protease inhibitor TIMP in tumor and endothelial cells [93]. Furthermore, recent work has uncovered an intricate new mechanism through which TGF-β acts in concert with oncogenic Ras to antagonize the p63-metastasis protective function [94]. p63 inhibition requires the combined action of Ras-activated mutant p53 and TGFβ-induced Smads, mechanistically involving the formation of a p63-Smads-mutant p53 ternary complex. Remarkably, two of the key downstream targets (*cyclin G2 and SHARP1*) of p63 are prognostic tools for breast cancer metastasis.

### 5.4. MicroRNAs (miRNAs) in Regulation of BCSCs

**Figure 1.**
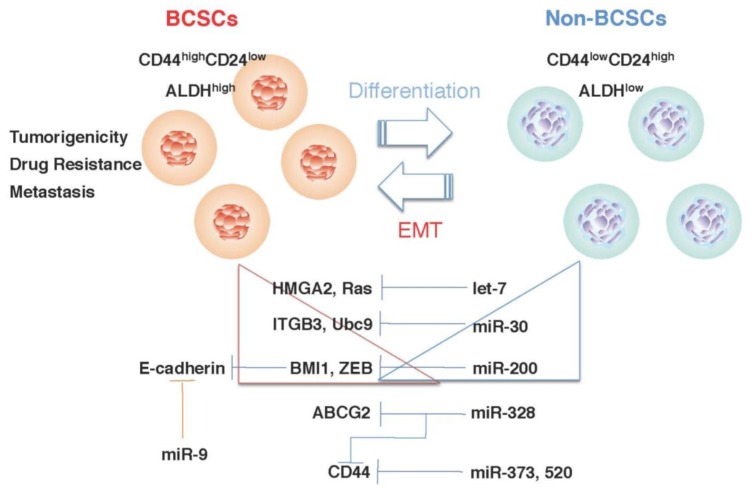
miRNA regulates BCSC phenotypes. Let-7 [61], miR-30 [102], and miR-200 [62] may regulate BCSC phenotypes by modulating the expression of their target genes. The expression of let-7, miR-30, and miR-200 is remarkably reduced in BCSCs, progressively increased with the differentiation of BCSCs, and inversely correlated with the expression of their target genes. miR-9 [101], miR-328 [67], miR-373 and miR-520c [103] also regulate BCSC phenotypes.

The emergence of miRNAs as important players in breast cancer has led researchers to explore the molecular mechanism that regulates BCSC phenotypes [61-63]. Some miRNAs play important roles in the phenotype formation of BCSCs, and multiple targets of these miRNAs have been identified [95,96] (Figure 1). Let-7 was originally identified in the nematode Caenorhabditis elegans [97,98]. Subsequently, the conservation of the let-7 sequence and its function in mammals were reported. Let-7 was one of the most consistently and significantly reduced miRNAs in many types of tumors, and downreglation of its expression was associated with the progression of tumor malignancy [99]. Recent studies have shown that let-7 suppresses self-renewal in both normal cells and BCSCs. Let-7 also regulates BCSC phenotypes such as chemoresistance and asymmetric cell division [61]. Several oncogenes, including c-Myc, Ras, HMGA2, cyclin D, and IL-6, have been reported as the targets of let-7 [61,63,99]. HMGA2 and Ras are highly expressed in BCSCs and remarkably repressed in non-BCSCs [61]. A comprehensive pathway involved in let-7 and lin-28 promotes the tumor metastasis and BCSC-induced inflammatory response [63]. Lin-28 is a highly specific embryonic stem cell marker and behaves as a negative regulator of let-7. Let-7 also directly represses lin-28 expression in ES and cancer cells [100].

In a comparison of the miRNA expression profiles of BCSCs and non-CSCs from human breast tumor samples, some miRNAs were upregulated and downregulated in BCSCs [62]. Three clusters, miR-200c-141, miR-200b-200a-429, and miR-183-96-182, were downregulated in human BCSCs [62]. Further study demonstrated that miR-200c regulated BCSC phenotypes by regulating the expression of BMI1, a member of the polycomb group of proteins, and acted as a negative regulator of stem cell phenotypes such as apoptosis, senescence, and differentiation [62].

miR-9, which is upregulated in breast cancer cells, directly targets CDH1, the E-cadherin-encoding messenger RNA, and also promotes cell motility and invasiveness [101]. miR-9-mediated E-cadherin downregulation results in the activation of beta-catenin signaling, which contributes to the upregulated expression of the gene encoding vascular endothelial growth factor (VEGF), which increases tumor angiogenesis [101]. Expression of miR-9 is activated by MYC and MYCN, both of which directly bind to the mir-9-3 locus. Significantly, in human cancers, miR-9 levels correlate with MYCN amplification, tumor grade, and metastatic status [101].

Thus, the dysregulation of miRNA is responsible for the BCSC phenotypes in the malignant progression of breast cancer, and the elucidation of the precise molecular mechanism may be the next step toward a more complete understanding of the regulatory networks underlying BCSC phenotypes, including metastasis.

## 6. CSC-Related Therapy

The development of therapies against CSCs is challenging because both bulk tumor cells and CSCs must be eliminated. As CSCs are molecularly distinct from bulk tumor cells, one can target their activity by exploiting the molecular differences [76,104-107]. For instance, cell surface marker expression could be used for antibody-directed therapy to target proteins, such as CD133, CD44, or ABC transporters, which are ATP-dependent drug efflux pumps [76,104-106]. A recent study identified ribophorin II (RPN2), part of an *N*-oligosaccharyl transferase complex, as a novel regulator of drug resistance via the regulation of the *N*-linked glycosylation of ABCB1 (P-glycoprotein) in breast cancer [107]. The downregulation of RPN2 most efficiently induces apoptosis of drug-resistant breast cancer cells in the presence of docetaxel. However, prior to developing an anti RPN2 therapy against cancer stem cells, the safety of the effect of RPN2 silencing in normal tissue stem cells should be tested.

Another strategy to selectively target CSCs is high-throughput compound screening. In one study, the antibiotic salinomycin preferentially killed BCSCs [7]. Interestingly, salinomycin induced the differentiation of BCSCs *in vivo*, as assessed by increased E-cadherin and reduced vimentin expression [7]. Furthermore, a recent study showed that salinomycin effectively reduced the number of P-glycoprotein expressing cells [108]. The activation of AMP-activated kinase with the diabetes drug metformin also resulted in the selective killing of CSCs in combination with chemotherapy [109]. This finding suggests that targeting mTOR activity, which is negatively regulated by AMP-activated kinase, may be a strategy to block BCSC phenotypes [110,111].

Recent studies have shown that oncolytic viruses seem to be well suited to eliminate CSCs because the viruses are cytotoxic and are not subject to drug efflux such as ABC transporters and defective apoptotic signaling [112]. These viruses are emerging as novel tools for cancer therapy, and several are already in clinical trials [113,114]. Virotherapy can also be utilized to sensitize tumor cells to radiation and chemotherapy and as tools for immunotherapy [115]. Thus, oncolytic viruses have significant advantages for improved treatment options for patients. In breast cancer, capsid-modified adenovirus vectors and reovirus vectors are effective for the elimination of BCSCs [116,117]. A recent study showed that BCSCs had dysregulated innate immune responses caused by impaired trafficking of toll-like receptor 9 (TLR9) and cofactor MyD88 and the absence of TLR2, resulting in dysfunctional virus recognition. These defects induced the dysfunctional induction of the type I interferon (IFN) response in BCSCs, leading to permissivity to the oncolytic adenovirus [118].

## 7. Conclusions

As accumulating evidence demonstrates that cancers are heterogeneous, clinical oncologists and cancer researchers need to consider which cancer cells have the potential to contribute to disease progression, including drug resistance and metastasis. Moreover, in breast cancer, recent studies have shown that non-CSCs acquire the CSC phenotype through EMT. Several key signaling pathways contribute to this process, namely, TGF-β and Wnt, known inducers of EMT and promoters of stem cell maintenance. The microRNAs also play critical roles in these processes, and the dysregulation of microRNA expression is likely to be a major contributing factor in CSC phenotypes. Therefore, targeting EMT pathways and CSC maintenance is a promising therapeutic strategy, one that seems to be feasible since several studies have successfully shown that pharmacological agents can modulate the differentiation state of a tumor. Thus, ‘differentiation-inducing’ agents such as salinomycin or metformin may have therapeutic value. The use of a natural miRNA to suppress the CSC phenotypes also promises to lead to a new therapeutic strategy for the treatment of CSCs.

Finally, the CSC phenotypes, which lead to high tumorigenicity, drug resistance, and metastasis, are responsible for many problems affecting cancer patients. Therefore, understanding and overcoming the CSC phenotypes will improve conventional cancer therapy.

## Figures and Tables

**Table1.** Representative Cell Surface Markers for Human Cancer Stem Cells (CSCs).

**Cancer Type**	**Cell Surface Markers**	**Ref.**
AML	CD34^+^CD38^-^	[6]
Breast Cancer	ESA^+^/CD44^+^/CD24^−/low^ ALDH1	[5] [26]
Glioma	CD133	[9,10]
Colon Cancer Metastatic Colon Cancer	CD133 CD133^+^/CD26^+^	[11,12] [27]
Melanoma	CD20 CD271	[13] [14]
Pancreatic Cancer Metastatic Pancreatic Cancer	ESA^+^/CD44^+^/CD24^+^ CD133^+^/CXCR4^+^	[16] [15]
Prostate Cancer	CD44^+^/α_2_β_1_^+^/CD133^+^	[17]
Ovarian Cancer	CD44^+^/CD117^+^ CD44^+^/MYD88^+^	[28] [29]
Hepatic Cancer	EpCAM	[25]
Lung Cancer	CD133	[20]
Gastric Cancer	CD44	[21]

AML: Acute myeloid leukemia; CXCR4: Chemokine receptor 4; ESA: Epithelial surface antigen; ALDH: Aldehyde dehydrogenase 1A1; α_2_β_1_: Integrin α_2_β_1_; MYD88: Myeloid differentiation primary response protein Myd88

**Table 2.** miRNAs Regulate the CSC Phenotypes.

**Cancer Type**	**microRNA**	**Potential Targets**	**Ref.**
Breast Cancer	let-7 miR-200c let-7 miR-200b	H-Ras and HMGA2 BMI1 IL-6 Suz12	[61] [62] [63] [64]
Glioblastoma	miR-199b-5p miR-34a miR-328	HES1 c-Met, Notch1, and Notch2 ABCG2	[65] [66] [67]
Medulloblastoma	miR128a	BMI1	[68]
Hepatic Cancer	miR-181	CDX2, GATA6, and NLK	[69]
Pancreatic Cancer	miR-34s	Bcl2 and Notch	[70]
Osteosarcoma and Colon Cancer	miR-140 miR-215	HDAC4 DHFR and Thymidylate synthase	[71] [72]

HMGA2: High mobility group AT-hook2; BMI1: BMI1 Polycomb ring finger oncogene; IL-6: Interleukin 6; SUZ12: Suppressor of zest 12 homolog; HES1: Hairy and enhancer of split 1; CDX2: Caudal type homeobox 2; GATA6: Gata binding protein 6; NLK: Nemo-like kinase 6; HDAC4: Histone deacetylase 4; DHFR: Dihydrofolate reductase
